# Association between physical activity and comorbid anxiety/depression in 46 low- and middle-income countries

**DOI:** 10.1016/j.jad.2022.10.002

**Published:** 2023-01-01

**Authors:** Ruimin Ma, Eugenia Romano, Davy Vancampfort, Joseph Firth, Brendon Stubbs, Ai Koyanagi

**Affiliations:** a Department of Psychological Medicine, Institute of Psychiatry, Psychology and Neuroscience (IoPPN), King's College London, London SE5 8AB, UK; b KU Leuven Department of Rehabilitation Sciences, Leuven 3000, Belgium; c University Psychiatric Centre KU Leuven, Kortenberg 3000, Belgium; d Division of Psychology and Mental Health, University of Manchester, Manchester M13 9PL, UK; e NICM Health Research Institute, Western Sydney University, Westmead 2751, Australia; f Physiotherapy Department, South London and Maudsley NHS Foundation Trust, Denmark Hill, London SE5 8AZ, UK; g Research and Development Unit, Parc Sanitari Sant Joan de Déu, Universitat de Barcelona, Fundació Sant Joan de Déu, CIBERSAM, ISCIII, 08830 Barcelona, Spain; h Institució Catalana de Recerca i Estudis Avancats (ICREA), Pg. Lluis Companys 23, 08010 Barcelona, Spain

**Keywords:** Physical activity, Depression, Anxiety, Low- and middle-income countries

## Abstract

**Background:**

Evidence on the association of low physical activity (PA) with depression or anxiety is well established. Yet, evidence on the association between PA and comorbid anxiety/depression remains scarce, especially in low- and middle-income countries (LMICs). Thus, this study explored this relationship among adults aged ≥18 years from 46 LMICs.

**Methods:**

Cross-sectional, community-based data were analyzed from the World Health Survey (WHS). Multivariable multinomial logistic regression analysis was conducted to examine the association between low PA and comorbid anxiety/depression with no anxiety or depression as the base category.

**Results:**

237,023 participants [mean (SD) age = 38.4 (16.0) years; 50.8 % female] were included in the analysis. Low PA was significantly associated with depression alone (OR = 1.33; 95%CI = 1.12–1.57) and anxiety alone (OR = 1.37; 95%CI = 1.23–1.53), while the OR was highest among those with comorbid anxiety/depression (OR = 1.75; 95%CI = 1.52–2.01).

**Conclusion:**

Low PA is associated with particularly increased odds for comorbid anxiety/depression. Increasing PA may have a beneficial effect on the prevention of comorbid anxiety/depression. However, future longitudinal research establishing the direction of this relationship is warranted.

## Highlights

• To explore the association between physical activity and comorbid depression/anxiety
• Low physical activity was associated with depression alone and anxiety alone.
• Low physical activity was associated with highest odds for comorbid depression/anxiety.

## 1 Introduction

Depression and anxiety are among the most commonly reported mental disorders worldwide (Wittchen and Jacobi, 2005). A report of the World Health Organization estimated that 4.4 % of the global population suffered from depressive disorder, and 3.6 % from anxiety disorder in 2015 (World Health Organisation, 2017). Moreover, according to the same report, depression and anxiety led to respectively over 50 million and 24.6 million years lived with disability, with most of this burden occurring in low- and middle-income countries (LMICs) (World Health Organisation, 2017). This is even more concerning when considering the comorbidity of these two conditions, with comorbid depression and anxiety occurring in up to 25 % of general practice patients. Indeed, significant anxiety is present in up to 85 % of patients with depression, while depression is present in about 90 % of patients with anxiety disorder (Tiller, 2013). Comorbid anxiety/depression is of particular concern not only because it can have a strong impact on the social and occupational functioning of a person (Belzer and Schneier, 2004), as well as on their physical and psychological quality of life (Zhou et al., 2017), but also because comorbid anxiety/depression is generally associated with a poor prognosis, higher functional disability and a more frequent use of health services (Tiller, 2013). In fact, there is evidence that patients with anxiety disorder and comorbid depression report an increased severity and chronicity of the condition, a higher likelihood of substance abuse, and a higher risk of suicide compared to patients with anxiety alone. Furthermore, patients with depression and comorbid anxiety also report worse response to medication and a poorer recovery from the index episode compared to those with depression alone (Pollack, 2005). Overall, the presence of depressive/anxiety comorbidity is associated with greater chronicity, an overall slower recovery, and a higher frequency of symptom recurrence (Hirschfeld, 2001). Finally, the specific co-occurrence of anxiety and depression has also been associated with a higher likelihood to develop certain several physical conditions, such as arthritis, chronic pain, and heart diseases (Scott et al., 2007), with a particularly elevated risk of mortality among patients with heart diseases (Doering et al., 2010; Watkins et al., 2013). Finally, there is evidence from data from LMICs that the co-occurrence of depression and anxiety is associated with a 30 % increased risk of premature mortality (Wu et al., 2020). Overall, this represents not only a burden for the individual, but for the socioeconomic system as well.

In low-income countries and middle-income countries respectively, about 76 % and 85 % of people with severe mental disorders receive no treatment for their disorders, and the yearly expenses for mental health are less than US$ 0.25 per person in these areas, where there is also a severe scarcity of healthcare personnel and resources (World Health Organization, 2013). Overall it is estimated that, while the costs to scale up treatments for depression and anxiety in LMICs could be high, this investment would result in improved health and increased economic productivity gain for these areas (Chisholm et al., 2016).

Physical activity (PA) could be an effective and low-cost intervention for improving mental health (Firth et al., 2020), especially in LMICs where there are economic constraints. Overall, literature suggests that PA could be a promising treatment for anxiety disorders (Kandola et al., 2018; Stubbs et al., 2017), even with moderate exercising such as walking (Merom et al., 2008). Moreover, a systematic review suggested that PA can have a strong beneficial effect on depression, comparable to the effects of antidepressant treatments (Dinas et al., 2011; Schuch et al., 2016), and a study observing the effects of aerobic and resistance training found that they both were effective in lowering the risk of developing depressive symptoms, as well as co-occurring depression and anxiety (Oftedal et al., 2019). Similarly, there is evidence from high-income countries on the association of PA patterns with the co-occurrence of anxiety and depression (Forte et al., 2020; Hiles et al., 2017), as well as data on how PA can have a positive effect on comorbid anxiety/depression, comparable to other kinds of psychological treatment (Ólafsdóttir et al., 2018).

There is a high co-morbidity and associated disability and premature mortality risk (Wu et al., 2020) and suboptimal treatment of comorbid anxiety and depression in LMICs (World Health Organization, 2013; Yatham et al., 2018). Different levels of knowledge regarding the benefits of PA (Pengpid et al., 2015), occupational and socio-cultural structures, methods of transportation, and environmental factors (e.g., safety, climate) (Atkinson et al., 2016) are also evident in this part of the world compared to high-income settings. However, to date, evidence supporting the association of PA and comorbid depression and anxiety in LMICs remains scarce. Two recent cross-sectional studies, one conducted in Southwest China (Liu et al., 2021) and one in Brazil (Schuch et al., 2020), supported a negative association between PA and co-occurring depressive and anxiety symptoms. Yet, both studies are single-country countries, and there are currently no multicountry studies on this topic from LMICs. Therefore, the aim of this study is to explore the relationship between PA and comorbid anxiety/depression among a sample of adults aged ≥18 years from 46 LMICs.

## 2 Methods

The World Health Survey (WHS) was a cross-sectional survey carried out in 70 countries in 2002–2004. Single-stage random sampling was undertaken in 10 countries, while stratified multi-stage random cluster sampling was conducted in 60 countries. Survey details are available elsewhere (Ustun et al., 2003). In brief, individuals with a valid home address aged ≥18 years were eligible to participate. Therefore, populations such as homeless people and institutionalized individuals were not included in the survey. Kish tables were used so that all household members had an equal chance of being selected. The questionnaire was subject to standard translation procedures to ensure comparability. Information was obtained through face-to-face interviews and telephone interviews conducted by trained interviewers. Across all countries, the individual response rate was 98.5 % (Nuevo et al., 2012). To adjust for non-response, sampling weights were generated using the population distribution as reported by the United Nations Statistical Division. Ethical approval for the survey was provided by ethical boards at each study site. All participants gave their informed consent.

### 2.1 Physical activity

PA was assessed using the short form of the International Physical Activity Questionnaire (IPAQ) (Craig et al., 2003), in which respondents are asked to report the number of days and the duration of the vigorous, moderate, and walking activities they undertook during the last 7 days. Show-cards illustrating different types of vigorous and moderate PA were presented to the respondents in addition to brief explanations on what was meant by vigorous and moderate activity. The summary indicator of the IPAQ was used to categorize the overall population into three levels of PA: low, moderate, or high. The moderate level nominally indicated meeting any of the following three criteria: (a) 3 days of vigorous activity of at least 20 min/day; (b) 5 days of moderate-intensity activity or walking of >30 min/day for >10 min at a time; or (c) 5 days of any combination of walking, moderate-intensity or vigorous-intensity activities achieving at least 600 MET-minutes/week. High levels of PA are defined as meeting either of two criteria: (a) vigorous-intensity activity on >3 days/week and accumulating at least 1500 MET-minutes/week; or (b) >5 days of any combination of walking, moderate-intensity, or vigorous-intensity activities achieving at least 3000 MET-minutes/week. One MET is defined as 1 kcal/kg/h and is more or less equivalent to the energy cost of sitting quietly (Craig et al., 2003). Those who neither meet the moderate nor high criteria were categorized in the low level PA group.

### 2.2 Anxiety and depression

Anxiety was assessed by the question ‘Overall in the past 30 days, how much of a problem did you have with worry or anxiety’ with answer options being none, mild, moderate, severe, and extreme. In accordance with previous WHS publications, those who answered severe and extreme were considered to have anxiety (Koyanagi and Stickley, 2015; Wong et al., 2013). Past 12-month depression was based on duration and persistence of depressive symptoms. Algorithms based on the DSM-IV (American Psychiatric Association, 1994) used in previous WHS publications were employed (Cifuentes et al., 2008; Loerbroks et al., 2012). Respondents were first asked five questions. Those who answered ‘Yes’ to four of them were considered as possibly having depression or a major depressive episode. Specifically, respondents were asked: “During the last 12 months have you ever experienced…”: (a) A period lasting several days when you felt sad, empty or depressed? (b) A period lasting several days when you lost interest in most things you usually enjoy such as hobbies, personal relationships or work? (c) A period lasting several days when you have been feeling your energy level decreased or that you were tired all the time? (d) Did you lose your appetite? (e) Did you notice any slowing down in your thinking?’. Among those with possible depression, individuals who further responded ‘Yes' to both of the following questions were classified as having depression: (a) Was this period for more than 2 weeks? (b) Was this period most of the day, nearly every day?

We created a four-category variable on anxiety/depression categories which included the following: (a) no anxiety, no depression; (b) depression without anxiety; (c) anxiety without depression; (d) anxiety and depression. Furthermore, a dichotomized variable of comorbid anxiety/depression (Y/N) was also created and used in country-wise analyses.

### 2.3 Control variables

The control variables included age (years), sex, education, and wealth. Education was categorized as: no formal education, primary education, secondary or high school completed, or tertiary education completed. Principal component analysis based on 15–20 assets was conducted to create country-wise wealth quintiles.

### 2.4 Statistical analysis

The statistical analysis was done with Stata 14.1 (Stata Corp LP, College station, Texas). Of the 69 countries for which data were publically available, 10 countries ((Austria, Belgium, Denmark, Germany, Greece, Guatemala, Italy, Netherlands, Slovenia, UK) with no sampling information were excluded. Country-income levels were based on the World Bank classification at the time of the survey (2003). We subsequently deleted 10 high-income countries (France, Finland, Ireland, Israel, Luxembourg, Norway, Portugal, Spain, Sweden, United Arab Emirates) as the focus of the study was on LMICs. Finally, Turkey was deleted due to lack of information on education, while Latvia and Morocco were also omitted due to lack of data on PA. All data were nationally representative with the exception of Russia, India, China, Comoros, Ivory Coast, and Republic of Congo.

Multivariable multinomial logistic regression analysis was conducted to assess the association between low PA (exposure) and anxiety/depression categories (outcome) using the overall sample. Next, we conducted country-wise binary logistic regression analysis with low PA as the exposure and comorbid anxiety/depression as the outcome. Furthermore, to assess whether there is between-country heterogeneity in the association between low PA and comorbid anxiety/depression, the Higgins's *I*^*2*^ statistic was calculated. This represents the degree of heterogeneity that is not explained by sampling error with a value of <40 % often considered as negligible and 40–60 % as moderate heterogeneity (Higgins and Thompson, 2002). A pooled estimate was obtained by combining the estimates for each country into a random effect meta-analysis.

The regression analyses were adjusted for age, sex, education, wealth, and country with the exception of the country-wise analysis which was not adjusted for country. Adjustment for country was done by including dummy variables for each country as in previous WHS publications (Koyanagi et al., 2017; Stubbs et al., 2016). The sample weighting and the complex study design were taken into account in all analyses. Results from the logistic regression models are presented as odds ratios (ORs) with 95 % confidence intervals (CIs). The level of statistical significance was set at *P* < 0.05.

## 3 Results

**Fig. 1:**
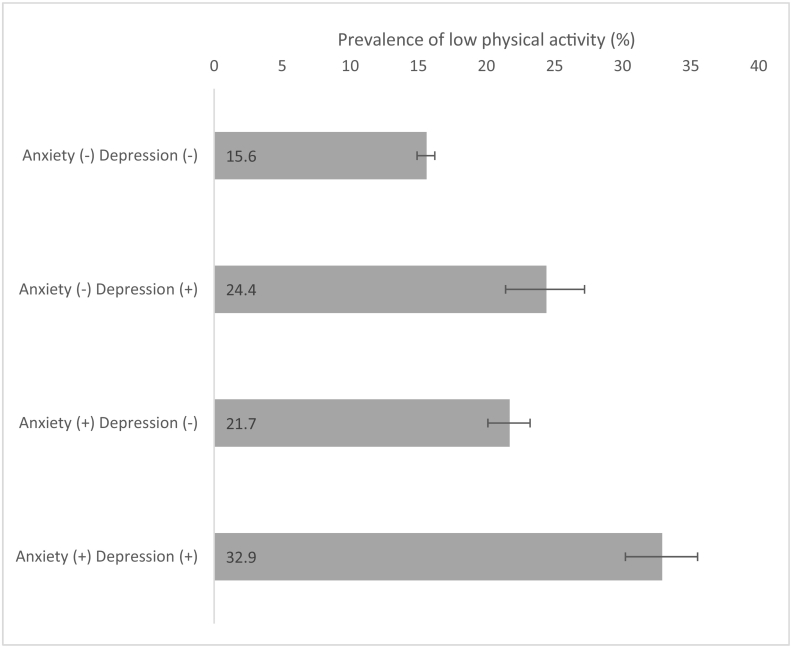
Prevalence of low physical activity by anxiety/depression categories. Bars denote 95 % confidence interval.

**Fig. 2:**
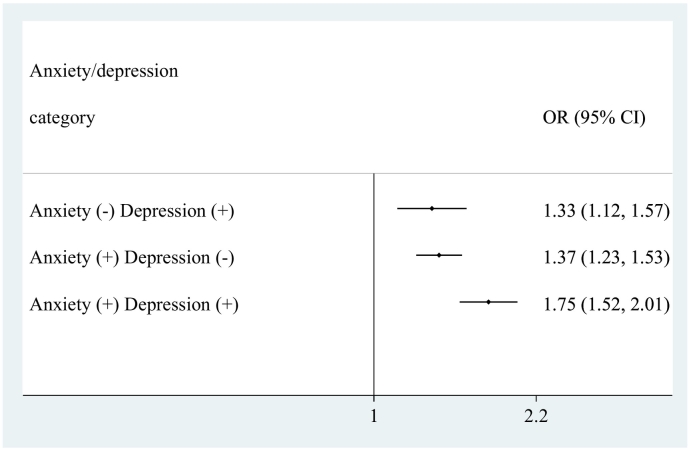
Association between low physical activity and anxiety/depression categories (outcome) estimated by multivariable multinomial logistic regression. Abbreviation: OR Odds ratio; CI Confidence interval. Base category is Anxiety (−) Depression (−). Model is adjusted for age, sex, education, wealth, and country.

**Fig. 3:**
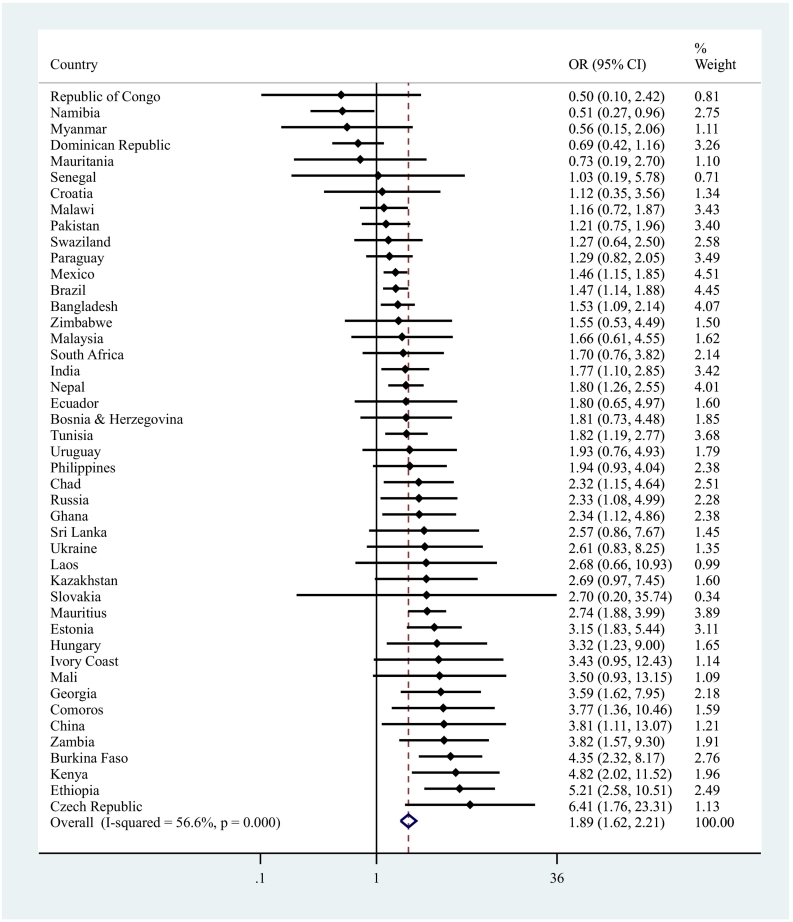
Country-wise association between low physical activity and comorbid anxiety/depression (vs. no comorbid anxiety/depression). Abbreviation: OR Odds ratio; CI Confidence interval. Models are adjusted for age and sex. Outcome is comorbid anxiety/depression. Overall estimate was based on meta-analysis with random effects. Estimates from Vietnam could not be obtained because the prevalence of comorbid anxiety/depression was extremely low.

The final sample consisted of 237,023 individuals aged ≥18 years from 46 LMICs. The mean (SD) age was 38.4 (16.0) years while 50.8 % were females. Overall, the prevalence of low PA was 17.4 %, while the prevalence of depression only, anxiety only, and comorbid anxiety/depression were 4.0 %, 8.2 %, and 2.8 %, respectively. The sample size of the included countries ranged from 949 (Czech Republic) to 38,746 (Mexico) (Table 1). The prevalence of low PA ranged from 5.1 % (Comoros) to 64.6 % (Mauritania), and the prevalence of comorbid anxiety/depression ranged from 0.01 % (Vietnam) to 9.00 % (Brazil). Among the four categories of anxiety/depression, the prevalence of low PA was highest among those with comorbid anxiety/depression (32.9 %) and this was much higher than in those without anxiety or depression (15.6 %) (Fig. 1). The association between low PA and anxiety/depression categories estimated by multivariable multinomial logistic regression with no anxiety and no depression as the base category is shown in Fig. 2. Low PA was significantly associated with depression alone (OR = 1.33; 95%CI = 1.12–1.57) and anxiety alone (OR = 1.37; 95%CI = 1.23–1.53), while the OR was highest among those with comorbid anxiety/depression (OR = 1.75; 95%CI = 1.52–2.01). In order to assess whether low PA is associated with a significantly higher odds for anxiety/depression comorbidity than depression alone or anxiety alone, we changed the base category. The results of this analysis showed that low PA is associated with 1.32 (95%CI = 1.07–1.62) and 1.27 (95%CI = 1.08–1.49) times higher odds for anxiety/depression comorbidity when the base category was depression alone and anxiety alone, respectively. The results of the country-wise analysis are shown in Fig. 3. Overall, low PA was associated with a 1.89 (95%CI = 1.62–2.21) times higher odds for comorbid anxiety/depression with a moderate level of between-country heterogeneity (*I*^*2*^ = 56.6 %).

**Table 1:** Sample size and prevalence of low physical activity and comorbid anxiety/depression by country.

Country	N	% Low PA	% Comorbid anxiety/depression
Bangladesh	5942	16.5	4.44
Bosnia & Herzegovina	1031	17.7	2.38
Brazil	5000	29.1	9.00
Burkina Faso	4948	8.4	1.71
Chad	4870	21.1	1.05
China	3994	11.4	0.28
Comoros	1836	5.1	2.16
Croatia	993	10.9	2.59
Czech Republic	949	13.4	2.18
Dominican Republic	5027	42.2	2.54
Ecuador	5675	22.7	1.62
Estonia	1020	7.0	2.13
Ethiopia	5089	5.4	2.44
Georgia	2950	10.5	1.78
Ghana	4165	13.0	1.43
Hungary	1419	11.1	1.55
India	10,687	14.5	3.47
Ivory Coast	3251	15.4	0.64
Kazakhstan	4499	13.6	1.75
Kenya	4640	9.6	1.55
Laos	4988	13.2	0.23
Malawi	5551	12.1	1.94
Malaysia	6145	20.2	0.41
Mali	4886	14.7	1.52
Mauritania	3902	64.6	0.77
Mauritius	3968	18.5	4.50
Mexico	38,746	18.0	1.64
Myanmar	6045	11.7	0.20
Namibia	4379	40.2	1.82
Nepal	8820	9.3	4.13
Pakistan	6501	21.1	1.46
Paraguay	5288	20.5	2.42
Philippines	10,083	7.9	0.68
Republic of Congo	3075	27.9	1.65
Russia	4427	10.0	2.04
Senegal	3461	18.0	1.44
Slovakia	2535	11.1	0.67
South Africa	2629	47.1	1.94
Sri Lanka	6805	11.4	0.58
Swaziland	3117	52.6	5.41
Tunisia	5202	16.3	3.10
Ukraine	2860	6.2	3.21
Uruguay	2996	28.2	1.53
Vietnam	4174	9.5	0.01
Zambia	4165	11.1	0.83
Zimbabwe	4290	19.4	1.38

Abbreviation: PA Physical activity.

## 4 Discussion

### 4.1 Main findings

To the best of our knowledge, this study is first multicountry study investigating the association between PA and comorbid depression and anxiety in LMICs. The association between low PA and comorbid anxiety/depression was particularly pronounced with this association being significantly stronger than associations between low PA and depression alone or anxiety alone. There was a moderate level of between-country heterogeneity in the association between low PA and comorbid anxiety/depression.

### 4.2 Interpretation of findings

The current study is one of few existing studies examining the association between PA and comorbid depression and anxiety (Hiles et al., 2017; Oftedal et al., 2019; Liu et al., 2021; Schuch et al., 2020). For example, Oftedal et al. (2019) demonstrated a relationship of aerobic PA and of aerobic PA plus resistance training with depression, and comorbid depression and anxiety. However, this study only included middle-aged Australian women, and therefore the findings cannot be generalized to the wider population. More severe anxiety and depression symptoms, and increased odds of disorder onset and chronicity at two-year follow-up were also found to be associated with lower sports participation at baseline in the Netherlands (Hiles et al., 2017). Similarly, two recent studies conducted in China (Liu et al., 2021) and Brazil (Schuch et al., 2020), respectively, also found a negative association between PA and comorbid depressive and anxiety symptoms. In line with these previous findings, the current study further underscores a significant association between low PA and the co-occurrence of depression and anxiety in multiple LMICs.

A range of neurobiological and psychological mechanisms explaining the antidepressive and anxiolytic effect of PA have been proposed. For example, for people with depression, PA may promote regulation of hypothalamic-pituitary-adrenal (HPA) axis functioning through modulating the impact of chronic stress on hippocampal functions for people with depression (Archer et al., 2015). PA has also been found to be beneficial in improving difficulties in sleeping and fatigue, which are frequently associated with depression (Feng et al., 2014; Youngstedt, 2005). With regards to anxiety, not only does PA improve the functioning of certain brain regions in the hippocampus (Firth et al., 2018), which are thought to be related to anxiety and stressful situations (Revest et al., 2009), but PA also has anti-inflammatory properties, which have also been associated with symptoms of anxiety (Vogelzangs et al., 2013). However, the mechanisms by which PA may improve the symptoms of comorbid depression and anxiety specifically are rarely explored. While the mechanisms may be expected to be similar to those for anxiety or depression alone, it is also possible that PA may motivate other healthy lifestyle behaviors (e.g., healthy diet, abstinence from alcohol and illegal drugs), promote positive mood (Meyer et al., 2016) and social integration (Yi and Hwang, 2015) for people with comorbid depression and anxiety. It could also be that PA reduces the impact of other risk factors that are shared by depression and anxiety, for example irritability, which is associated with onset of several psychiatric disorders including major depressive disorder and generalized anxiety disorder (Stringaris et al., 2009).

We also found a moderate level of between-country heterogeneity in the association between PA and comorbid anxiety/depression. Although the reasons for this can only be speculated, it is possible that the content of PA differs by countries or that this may be reflecting differences in levels of health care. Future studies that examine the reasons for this between-country heterogeneity are warranted.

### 4.3 Public health and clinical implications

Our study is by far the largest and the first multicountry study on PA and comorbid anxiety/depression from LMICs, where frontline treatments for depression and anxiety (e.g., antidepressants, psychotherapy) are generally limited (World Health Organization, 2013). Furthermore, in LMICs, there is a high rate of out-of-pocket expenditures (Van Doorslaer et al., 2007), and a lack of access to easily accessible, low-cost, attractive, and safe PA facilities in- and outdoors (Bojorquez et al., 2021; Vancampfort et al., 2019). There has been a number of randomized controlled trials (RCTs) examining the effect of PA on mental health outcomes (Firth et al., 2020) and the majority were restricted to depression (Kruisdijk et al., 2019; Lewis et al., 2014; Ström et al., 2013) and anxiety (Powers et al., 2015; Rosenbaum et al., 2015) alone. The benefits of PA have been reported in some RCTs (Rosenbaum et al., 2015; Ström et al., 2013) but not others (Kruisdijk et al., 2019; Lewis et al., 2014). Stubbs et al.' (2017) meta-analysis suggests a high volume of evidence from RCTs supporting the benefits of PA on mental health symptoms. However, to our knowledge, no RCTs to date have investigated the efficacy of PA on the co-occurrence of depression and anxiety specifically. High quality RCTs are essential steps in understanding the role of PA on co-morbid mental health symptoms in clinical populations and the general population. Therefore, there is an urgent need for future well-designed and adequately powered RCTs to be conducted, in order to recognize the extent to which characteristics of PA with respect to its intensity, duration, frequency and type may alleviate or prevent co-occurring depression and anxiety. Multidisciplinary approaches involving psychiatrists, psychologists, sport trainers and other mental healthcare professionals are also recommended to facilitate the delivery of exercise interventions, either as an add-on treatment option or as an alternative for patients in LMICs where standard treatments for these conditions are not available (Stubbs et al., 2017). Despite the promising benefits of involvement of health professionals in high-income countries, an expert-led PA program may not always be available in LMICs and its sustainability has been questioned (Vancampfort et al., 2021). The involvement of lay health workers or non-professional community health workers that lead PA counselling programs may instead be a more feasible solution for LMICs (Lehmann and Sanders, 2007; Vancampfort et al., 2021).

### 4.4 Strength and limitations

The current study benefits from a number of strengths, including a large multi-national sample, and a specific focus on the comorbidity of depression and anxiety. However, the current study should also be interpreted in light of its limitations. First, this study is cross-sectional in nature, therefore, the direction of the relationships could not be determined. However, we may speculate that these relationships could be reciprocal over time. For example, certain symptoms of anxiety and depression (e.g., low motivation, fatigue and poor concentration) may induce a sedentary lifestyle, low sports participation, and PA levels (Hiles et al., 2017; Picorelli et al., 2014), and this may lead to further exacerbation of symptoms of depression or anxiety. A few longitudinal studies have examined these relationships (Hiles et al., 2017; Lindwall et al., 2011), and future research re-confirming the association between PA, depression, anxiety and most importantly comorbid depression and anxiety among LMICs through well-implemented longitudinal design is therefore encouraged. Second, anxiety was assessed by one question only and this question only asked anxiety symptoms within the past 30 days, which is not equivalent to a clinical diagnosis. However, the use of extreme categories to define anxiety is likely to have increased specificity. Finally, PA level was determined by self-report measures, hence our results could be limited by recall and reporting bias. Objective measures of PA, for example using an accelerometer, should be considered in future studies.

## 5 Conclusion

Previous studies have mostly focused only on the associations between PA with either anxiety alone or depression alone, whereas the results of this study suggest that PA may be particularly beneficial to improve the symptoms of comorbid anxiety/depression in LMICs, also given that the levels of PA among this population is particularly low. However, due to the cross-sectional nature of the current study, future longitudinal research examining the benefits of PA on the improvement of comorbid depression/anxiety over time is warranted to be able to make concrete recommendations. Along with conducting large-scale RCTs to confirm these effects, LMIC settings may benefit from adopting strategies to increase public awareness of PA in mental healthcare, along with investment in exercise interventions and prescription in the context of mental healthcare.

## CRediT authorship contribution statement

BS, AK, RM, ER designed the study. AK and BS acquired funding for the study. AK conducted the analysis with support from all co-authors. RM and ER drafted the first version and all authors (BS, AK, DV, JF) provided critical revisions and approved the final version.

## Funding

This research was supported by the 10.13039/501100000265Medical Research Council (Grant Reference: MC_PC_MR/T037806/1 and in part by the 10.13039/501100000272National Institute of Health Research using Official Development Assistance (ODA) funding (Grant: 17/63/130: NIHR Global Health Research Group: Improving Outcomes in Mental and Physical Multimorbidity and Developing Research Capacity (IMPACT) in South Asia at the University of York). BS is supported by a Clinical Lectureship (ICA-CL-2017-03-001) jointly funded by Health Education England (HEE) and the 10.13039/501100000272National Institute for Health Research (NIHR). Brendon Stubbs is part funded by the NIHR Biomedical Research Centre at South London and Maudsley NHS Foundation Trust. Brendon Stubbs also holds active grants with the Medical Research Council (GCRF and multimorbidity calls) and Guys and St Thomas Charity (GSTT). JF is supported by a University of Manchester Presidential Fellowship (P123958) and a UK Research and Innovation Future Leaders Fellowship (MR/T021780/1) and has received honoraria / consultancy fees from Atheneum, ParachuteBH and Nirakara, independent of this work.

## Sponsor's role

The funding resources had no involvement in the design, analysis or reporting of the results.

## Ethnical standards

The authors assert that all procedures contributing to this work comply with the ethical standards of the relevant national and institutional committees on human experimentation and with the Helsinki Declaration of 1975, as revised in 2008.

## Institutional review board statement

Ethical approval for the World Health Survey was provided by ethical boards at each study site. All participants gave their informed consent.

## Informed consent statement

Informed consent was obtained from all subjects involved in the World Health Survey.

## Conflict of Interest

All authors declare no conflicts of interest.

## Data Availability

The datasets generated and/or analyzed during the current study are publicly available in the WHO World Health Survey repository: https://apps.who.int/healthinfo/systems/surveydata/index.php/catalog/whs
